# Mucosal CD8 T Cell Responses Are Shaped by Batf3-DC After Foodborne *Listeria monocytogenes* Infection

**DOI:** 10.3389/fimmu.2020.575967

**Published:** 2020-09-11

**Authors:** Jessica Nancy Imperato, Daqi Xu, Pablo A. Romagnoli, Zhijuan Qiu, Pedro Perez, Camille Khairallah, Quynh-Mai Pham, Anna Andrusaite, Alberto Bravo-Blas, Simon W. F. Milling, Leo Lefrancois, Kamal M. Khanna, Lynn Puddington, Brian S. Sheridan

**Affiliations:** ^1^Department of Microbiology and Immunology, Center for Infectious Diseases, Stony Brook University Renaissance School of Medicine, Stony Brook, NY, United States; ^2^Department of Immunology, UConn Health, Farmington, CT, United States; ^3^Centro de Investigacion en Medicina Traslacional Severo Amuchastegui, Instituto Universitario de Ciencias Biomédicas de Córdoba, Córdoba, Argentina; ^4^Centre for Immunobiology, Institute of Infection, Immunity, and Inflammation, University of Glasgow, Glasgow, United Kingdom; ^5^The Beatson Institute for Cancer Research, Glasgow, United Kingdom; ^6^Department of Microbiology, New York University, New York City, NY, United States

**Keywords:** dendritic cells, CD8 T cells, intestine (small), *Listeria (L.) monocytogenes*, *Batf3*

## Abstract

While immune responses have been rigorously examined after intravenous *Listeria monocytogenes* (*Lm*) infection, less is understood about its dissemination from the intestines or the induction of adaptive immunity after more physiologic models of foodborne infection. Consequently, this study focused on early events in the intestinal mucosa and draining mesenteric lymph nodes (MLN) using foodborne infection of mice with *Lm* modified to invade murine intestinal epithelium (InlA^M^ *Lm)*. InlA^M^ *Lm* trafficked intracellularly from the intestines to the MLN and were associated with Batf3-independent dendritic cells (DC) in the lymphatics. Consistent with this, InlA^M^ *Lm* initially disseminated from the gut to the MLN normally in *Batf3*^–/–^ mice. Activated migratory DC accumulated in the MLN by 3 days post-infection and surrounded foci of InlA^M^ *Lm*. At this time *Batf3*^–/–^ mice displayed reduced InlA^M^ *Lm* burdens, implicating cDC1 in maximal bacterial accumulation in the MLN. *Batf3*^–/–^ mice also exhibited profound defects in the induction and gut-homing of InlA^M^ *Lm*-specific effector CD8 T cells. Restoration of pathogen burden did not rescue antigen-specific CD8 T cell responses in *Batf3*^–/–^ mice, indicating a critical role for *Batf3* in generating anti-InlA^M^ *Lm* immunity following foodborne infection. Collectively, these data suggest that DC play diverse, dynamic roles in the early events following foodborne InlA^M^ *Lm* infection and in driving the establishment of intestinal *Lm*-specific effector T cells.

## Introduction

*Listeria monocytogenes* (*Lm*) is a Gram-positive, intracellular pathogen found ubiquitously throughout the environment. As an enteric pathogen, *Lm* may lead to a mild gastrointestinal distress in healthy human hosts; however, those who are elderly, pregnant, or otherwise immunocompromised, and contract listeriosis risk the development of meningitis, sepsis, or stillbirths. In the United States, listeriosis is typically associated with mortality rates ranging 20–50% among hospitalized individuals (1). *Lm* was recently responsible for the largest foodborne outbreak in the world, in which over 1,000 people became ill and over 200 deaths occurred in South Africa as a result of contaminated meat (2). Consequently, *Lm* remains a major public health risk. *Lm* also has the ability to induce potent CD8 T cell responses and break tolerance to tumor antigens, making it a promising candidate as a vector for anti-cancer vaccines (3). Therefore, the potential use of *Lm* for the development and generation of mucosal vaccines against enteric pathogens or gastrointestinal cancers has engendered the evaluation of intestinal immune responses in the context of foodborne *Lm* infection or oral immunization.

The generation of robust adaptive immunity at intestinal inductive sites is dependent on interactions between professional antigen-presenting cells (APC) and T cells. Dendritic cells (DC) in particular survey tissues and phagocytose pathogens before transiting through afferent lymphatic vessels to draining lymph nodes, where antigen is presented to naïve T cells to elicit an effector T cell response (4). DC are a heterogenous population with discrete functions, ontogeny and anatomic compartmentalization (5). Lymphoid tissue-residing CD8α^+^ DC have been linked both developmentally and functionally to migratory CD103^+^ DC through their dependence on IRF8 and BATF3 for their development and the ability to cross-present antigens to CD8 T cells (5–7). Together, these Batf3-dependent DC are classified as type 1 conventional DC (cDC1) (8). Migratory small intestinal lamina propria (SI-LP) and MLN cDC1 retain the ability to induce the expression of the gut-homing markers CCR9 and α_4_β_7_ upon T cell activation, resulting in gut tropism and localization to the small intestines (9, 10). Meanwhile gut migratory, Irf4-dependent CD11b^+^ CD103^+^ DC (cDC2), have been implicated in T_H_17 cell differentiation (11, 12). Finally, a subset of migratory CD11b^+^ CD103^–^ DC are important in the differentiation of effector T cells that produce IFNγ and IL-17A (13, 14).

DC are necessary for the formation of cytotoxic responses to eliminate *Lm* after intravenous (i.v.) infection (15). After i.v *Lm* infection, splenic marginal zone B cells produce IL-10, which acts on CD169^+^ macrophages to promote bacterial uptake and survival (16). *Lm*-containing CD169^+^ macrophages trans-infect cDC1 to mediate *Lm* transport to the splenic T cell zones. Thus, CD169^+^ macrophages and cDC1 provide critical non-redundant roles to initiate infection and promote resolution (16–18). Mice lacking cDC1 are resistant to *Lm* infection with reduced pathogen burden but display diminished *Lm*-specific CD8 T cell responses (6, 19). However, restoration of pathogen burden by increasing the infection dose in these mice rescues the CD8 T cell response, revealing that cDC1 are not required for T cell responses to *Lm* after i.v. infection (17). Despite these findings, cDC2 appear to play minimal roles in the generation of protective immunity to *Lm*, as mice lacking cDC2 do not exhibit defective CD8 T cell responses (16). Although the roles of resident cDC1 and cDC2 after i.v *Lm* infection have been assessed, the functions of migratory cDC in the early establishment of *Lm* infection and the induction of T cell responses after foodborne infection have not been extensively studied (20). Some evidence has emerged from foodborne infection of a susceptible Balb/cBy model that suggests that most *Lm* replicates extracellularly (21). While these studies noted that only a minor amount of *Lm* is intracellular following foodborne infection, the intracellular fraction was necessary for invasion into the small intestines, as well as extraintestinal dissemination. Monocytes, which are critical for *Lm* containment, were identified as a major cell type associated with *Lm* (22). Intracellular bacteria could also be found within intestinal cDC (23). However, monocytes and *ex vivo* DC did not support intracellular growth suggesting these cells do not serve as a major niche needed to propagate *Lm* infection (21, 22).

In this study, foodborne infection with murinized *Lm* containing a mutation in its internalin A gene (further referred to as InlA^M^ *Lm*) that recapitulates physiologic infection in humans was used to investigate intestinal immune responses in Balb/c and B6 mice (24–26). After foodborne infection, InlA^M^ *Lm* transited intracellularly from the intestines to the MLN. However, maximal accumulation of bacteria in the MLN appeared dependent on *Batf3*, suggesting a role for cDC1 in this process. Finally, InlA^M^ *Lm*-specific CD8 T cell responses were diminished in *Batf3*^–/–^ mice and restoration of pathogen burden was unable to restore InlA^M^ *Lm*-specific CD8 T cell responses, suggesting a dynamic role for DC subsets in driving effector CD8 T cell responses after foodborne InlA^M^ *Lm* infection.

## Materials and Methods

### Mice

Balb/c and *Batf3*^–/–^ Balb/c mice were utilized for all experiments, unless otherwise noted. For experiments involving OT-I cell transfers and thoracic duct cannulations, C57BL/6 (B6) were used. *Batf3*^–/–^ and OT-I TCR transgenic *Rag1*^–/–^ CD45 congenic mice were bred in-house. For experiments assessing the role of IRF4-dependent DC after foodborne InlA^M^ *Lm* infection, *Itgax*-cre *Irf4*^fl/fl^ and littermate control *Irf4*^fl/fl^ mice (B6 background) were used. Both male and female mice 8–19 weeks of age were used. Mice were age- and sex-matched to controls from The Jackson Laboratory (Balb/c) or Charles River/NCI (B6). All mice were housed and treated in accordance with the Institutional Animal Care and Use Committee guidelines and approved by Stony Brook University and UConn Health. All survival surgeries were performed at the University of Glasgow, in accordance with the Animal Welfare Ethical Review Board. The Review Board and UK Home Office approved both the individual license (IBCA6BF4E) and project license (P64BCA712), which are compliant with the Animals (Scientific Procedures) Act 1986. This Act amended European Directive 2010/63/EU on the protection of animals used for scientific purposes.

### Foodborne InlA^M^ *Lm* Infection

For infections in Balb/c mice, InlA^M^ *Lm* (strain 10403s or EGDe as indicated) was utilized. Mice were deprived of food and water 4–6 h prior to infection, then housed individually and given ∼0.5 cm^3^ pieces of white bread inoculated with 2 × 10^9^ CFU InlA^M^ *Lm* in PBS (unless otherwise noted). For infections involving B6 mice, InlA^M^ *Lm* expressing a truncated ovalbumin (InlA^M^ *Lm*-OVA; strain 10403s) was used.

### Treatments

Mice received gentamicin (1 mg, subcutaneous) or an equal volume of PBS at 4 h post-infection (hpi), 20 mg streptomycin (oral gavage) at day -1, or 250 μg anti-Ly6G mAb (IA8) or control IgG2 at days -1, 0, 1, and 2 (intraperitoneal injection).

### Microscopy

At 3 days post-infection (dpi), MLN were harvested, fixed in paraformaldehyde-lysine-periodate (PLP) solution, washed with PBS, and dehydrated in 30% sucrose. MLN were snap-frozen in OCT compound (Tissue-Tek), and 30 μm sections were cut using a cryostat (Leica CM1850). Sections were fixed in acetone for 10 min and stained with primary antibodies diluted in PBS with 2% GS and 2% FCS for 1.5 h at room temperature, washed, and stained with secondary or fluorophore-conjugated antibodies diluted in PBS with 2% GS and 2% FCS for 1 h at room temperature. Images were acquired with an LSM780 (Carl Zeiss, Oberkochen, Germany) and processed with Imaris software (Bitplane, Belfast, United Kingdom). All antibodies used are listed in Supplementary Table 1.

### Enumerating Listeria Burden

Liver, spleen, and MLN were processed in 1% saponin. Small intestinal contents were flushed using RPMI containing 5% heat-inactivated bovine serum, homogenized using a GentleMACS (Miltenyi Biotec, Auburn, CA, United States), and lysed with 1% saponin. All suspensions were incubated at 4°C for at least 1 h. All bacterial burdens were plated onto Brain Heart Infusion (BHI) agar containing 200 μg/mL streptomycin, incubated at 37°C, and enumerated 24–48 h later.

### Dendritic Cell Isolation, Sorting, and Burden

At 3 dpi, MLN were harvested, pooled together, and digested with 100 U/mL collagenase 37°C for 30 min. MLN were crushed through a 70 μm filter, counted, and stained in PBS containing heat-inactivated bovine serum with fluorochrome-conjugated antibodies. DC subsets were sorted on a FACSAria (Becton, Dickinson and Company, Franklin Lakes, NJ, United States). Sorted subsets were directly plated onto BHI agar containing 200 μg/mL streptomycin to enumerate bacterial burden. All antibodies used are listed in Supplementary Table 1.

### Thoracic Duct Cannulation

Mesenteric lymphadenectomy (MLNx) was performed on 6-week old B6 males as outlined previously (27, 28). 6 weeks after MLNx, mice were infected with 2 × 10^9^ CFU InlA^M^ *Lm* (strain 10403s). At 1 dpi, mice received 200–300 μL of olive oil via oral gavage, to visualize the lymphatics. A polyurethane cannula (2Fr; Linton Instrumentation, Diss, United Kingdom) was surgically inserted into the thoracic duct. Lymph was collected into 1mL PBS containing 5,000 U/mL heparin sodium (Wockhardt United Kingdom, Wrexham, United Kingdom) on ice overnight. Collected lymph was stained using fluorochrome-conjugated antibodies and sorted on a FACSAria (Becton, Dickinson and Company, Wokingham, United Kingdom). Sorted DC were lysed with Triton X, plated directly onto BHI agar containing 200 μg/mL streptomycin to enumerate bacterial burden. For all surgeries, mice were anesthetized with isoflurane (Abbott Labs, Abbott Park, IL, United States).

### Lymphocyte Isolation and Flow Cytometry

Single-cell suspensions were prepared from the MLN and spleen by mechanical dissociation. Isolation of DC also utilized enzymatic processing with 100 U/mL collagenase at ambient temperature for 25 min and the addition of 0.1M EDTA prior to mechanical dissociation. For the spleen, red blood cells were lysed in RBC Lysis Buffer (BioLegend, San Diego, CA, United States). Intraepithelial lymphocytes (IEL) were isolated with dithioerythritol as described previously (29, 30). Cells were stained with fluorochrome-conjugated antibodies for 30 min at 4°C in the dark. Panels containing MHC class I tetramers were stained for 1 h in the dark at ambient temperature. Cells were fixed with 2% paraformaldehyde (PFA) for 20 min at 4°C in the dark. Samples were acquired on a LSRFortessa (Becton, Dickinson and Company, Franklin Lakes, NJ, United States), and analyzed with FlowJo (software version v10, Becton, Dickinson, and Company, Ashland, OR, United States). All antibodies used are listed in Supplementary Table 1.

### OT-I Transfer Experiments

For location of antigen presentation, 1 × 10^6^ CFSE-labeled CD45.1 OT-I cells were transferred into B6 mice at 1 dpi. 16 h later, MLN and spleen were harvested, processed, and cultured in complete RPMI containing 10 IU human IL-2 for 3 days at 37°C, 5% CO_2_, and CFSE dilution was analyzed as previously described (31, 32). For duration of antigen presentation, 1 × 10^6^ CFSE-labeled CD45.1 OT-I cells were transferred into infected B6 mice at the indicated times. 3 days after transfer, the MLN and spleen were harvested and processed to assess CFSE dilutions. All antibodies used are listed in Supplementary Table 1.

### Statistics

All statistical analyses were performed using Prism 8.3.1 (GraphPad).

## Results

### InlA^M^ *Lm* Disseminates Intracellularly From the Intestines to the MLN After Foodborne Infection

**FIGURE 1:**
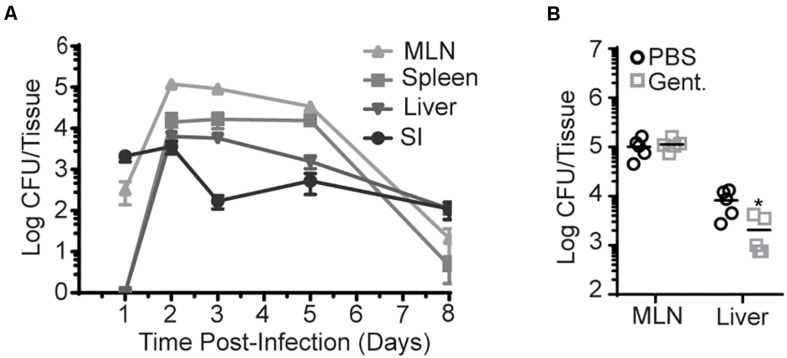
InlA^M^ *Lm* accumulation in the MLN is intracellular after foodborne infection. Bacterial burden was enumerated from the organs of Balb/c mice after oral infection with InlA^M^ *Lm* 10403s. **(A)** Bacterial burden was enumerated from the MLN, spleen, liver, and small intestines from 1-8 dpi after infection with 2 × 10^9^ CFU InlA^M^ *Lm*. 1 and 3 dpi burdens have been repeated at least 3 times. The complete time course has been performed 1 time in Balb/c mice and 1 time in B6 mice (Supplementary Figure S1A), and 1, 3, and 5 dpi have been depicted in multiple independent experiments reported herein. **(B)** Mice were foodborne infected with 2 × 10^9^ CFU InlA^M^ *Lm* and were treated with either gentamicin or PBS at 4 hpi. At 3 dpi, MLN and livers were harvested, and bacterial burdens were quantified. **(A)** Data depicts 5 mice per timepoint. **(B)** Data are representative of 2 independent experiments containing 5 mice per group. Statistics were determined by Mann-Whitney test: *p < 0.05.

Infection route plays an important role in bacterial invasion and dissemination. As such, InlA^M^ *Lm* dissemination and propagation were analyzed in different tissues after foodborne infection of Balb/c or C57BL/6 (B6) mice with InlA^M^ *Lm*-inoculated bread. One day after infection, bacteria were present in the intestine and MLN but were absent from the spleen or liver (Figure 1A and Supplementary Figure S1A). However, by 2 dpi, InlA^M^ *Lm* had disseminated to the liver and spleen. The bacterial burden peaked at 2–3 dpi in all tissues examined and was largely cleared between 5 and 8 dpi. The kinetic analysis of InlA^M^ *Lm* dissemination after foodborne infection of Balb/c and B6 mice suggests a stepwise pattern of dissemination in which InlA^M^ *Lm* initially mobilizes from the intestines to the draining MLN before dissemination to extraintestinal sites like the spleen and liver. No evidence emerged of a rapid and direct mobilization of InlA^M^ *Lm* to the liver. Thus, foodborne InlA^M^ *Lm* infection recapitulates the dissemination pattern expected for an intracellular pathogen acquired through the gastrointestinal tract.

To assess this more directly, gentamicin was administered to foodborne infected mice to kill extracellular InlA^M^ *Lm*. As gentamicin is unable to effectively enter cells at the concentration used, intracellular InlA^M^ *Lm* remains viable in its presence. Gentamicin was administered via subcutaneous injection to Balb/c mice 4 h post-foodborne InlA^M^ *Lm* infection to allow initial invasion, and bacterial burdens were enumerated at 3 dpi. Subcutaneous injection was chosen as gentamicin is not efficiently absorbed by the intestines (33). At an infection dose of 2 × 10^9^ CFU, the InlA^M^ *Lm* burden was unaffected in the MLN but decreased approximately fourfold in the liver after antibiotic treatment (Figure 1B). Thus, InlA^M^ *Lm* is protected from the effects of gentamicin by containment inside cells within the MLN, while a substantial proportion of *Lm* in the liver has an extracellular component to their lifecycle that promotes gentamicin susceptibility. Increasing the infection dose leads to more extracellular InlA^M^ *Lm* in the spleen and liver after i.v. *Lm* infection (34). To confirm gentamicin is effective in the MLN and determine whether increased infectious dose drives an extracellular phase after foodborne InlA^M^ *Lm* infection of resistant mice, mice were infected with 2 × 10^10^ CFU of InlA^M^ *Lm* prior to gentamicin treatment. In this context, gentamicin reduced the recoverable InlA^M^ *Lm* burden from the MLN and liver at 3 dpi (Supplementary Figure S1B). As neutrophils have been shown to be critical for clearance of extracellular InlA^M^ *Lm* but dispensable for control of intracellular *Lm*, the capacity of neutrophil depletion to impact bacterial burdens after foodborne InlA^M^ *Lm* was tested (34, 35). As such, mice were treated with neutrophil depleting anti-Ly6G mAb (IA8) after foodborne infection. Consistent with the results after gentamicin treatment, neutrophils were dispensable in the MLN but required in the liver for InlA^M^ *Lm* control (Supplementary Figure S1C). This pattern of predominately intracellular compartmentalization in lymphoid tissues and extracellular compartmentalization in the liver is consistent with *Lm* replication after i.v. infection. Collectively, these data support the notion that bacteria shuttles intracellularly to the MLN after foodborne infection of Balb/c mice (21). It should be noted that increasing the infection dose does not lead to more extracellular InlA^M^ *Lm* after foodborne infection of susceptible Balb/cBy mice (21). This model of foodborne infection also showed that a large proportion of gastrointestinal InlA^M^ *Lm* is extracellular (21). At similar infectious doses of 2 × 10^9^ CFU, Balb/cBy mice had more replicative InlA^M^ *Lm* in the MLN and liver than either Balb/c or B6 mice (Supplementary Figure S1D). Balb/cBy were also more susceptible to foodborne InlA^M^ *Lm* infection as only 20% of mice survived until 7 dpi, while B6 and Balb/c mice fully recover at this dose (Supplementary Figure S1E). Taken in context together, these data suggest that the extracellular nature of InlA^M^ *Lm* infection of Balb/cBy mice may be associated with increased bacterial burden. Therefore, differences in bacterial burden and cellular compartmentalization among Balb/cBy and Balb/c mice could explain the differences noted between foodborne InlA^M^ *Lm* infection models.

### Migratory DC Are Activated and Accumulate Around Regions of InlA^M^ *Lm* Foci in the MLN After Foodborne Infection

**FIGURE 2:**
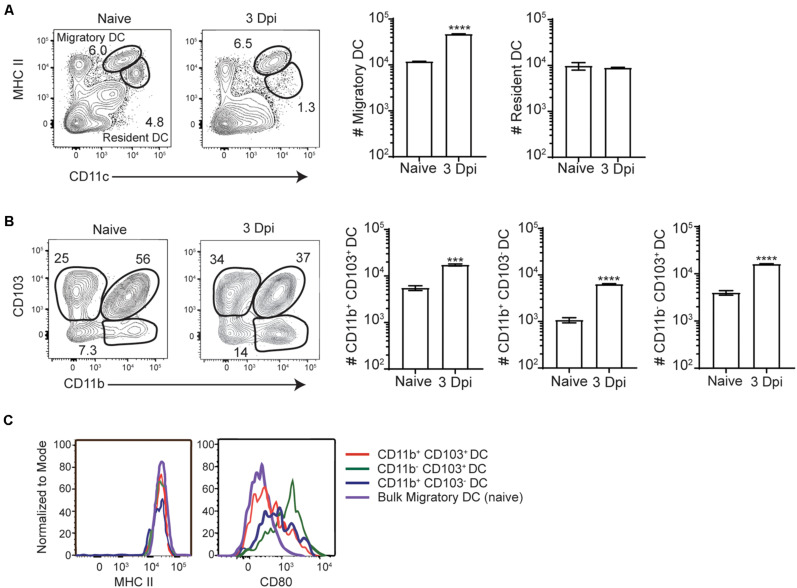
Migratory DC are activated and accumulate in the MLN after foodborne InlA^M^ *Lm* infection. MLN were harvested from Balb/c mice after foodborne infection with 2 × 10^9^ CFU InlA^M^ *Lm* 10403s and dendritic cells subsets were analyzed via flow cytometry. **(A)** Representative flow plots from the MLN of naïve and 3 dpi mice depicting the gating scheme used to identify migratory and resident DC subsets. The populations represented are gated on B220^–^ TCRβ^–^ single, live leukocytes. **(B)** Cell counts were calculated for migratory dendritic cell subsets at naïve and 3 dpi time points after infection. DC subsets were identified as noted previously, and cell numbers were calculated using cell frequencies and ViCell counts of total viable cells. **(C)** Representative histograms depicting CD80 and MHCII expression levels on migratory DC subsets. Cell subsets were gated as demonstrated in panels **(A,B)**. Data are representative of two independent experiments with 3–4 mice per group. Unpaired *T*-tests are reported: ***p < 0.001; ****p < 0.0001.

Intracellular bacterial accumulation in the MLN suggested that InlA^M^ *Lm* may transit to the MLN from the intestines via migratory innate immune cells after foodborne infection. While intestinal macrophages are thought to largely remain tissue-resident within the intestines, DC have been shown to readily migrate from the intestines to the MLN during normal homeostasis or after perturbations of homeostasis such as infection or inflammation (7, 36). As such, resident and migrant DC subsets were evaluated in the MLN of Balb/c mice at steady state and 3 days after foodborne InlA^M^ *Lm* infection. By 3 dpi, migratory (identified by MHCII^hi^ CD11c^+^) but not resident (identified by MHCII^+^ CD11c^+^) DC accumulated in the MLN of foodborne infected mice (Figure 2A). Migratory DC from the intestines are typically separated into 3 major subsets based on CD103 and CD11b expression (CD103^+^ CD11b^–^, CD103^+^ CD11b^+^, and CD103^–^ CD11b^+^) and each of these were increased in the MLN at 3 dpi (Figure 2B). While all 3 migratory subsets expressed high levels of MHCII, migratory CD103^+^ CD11b^–^ cDC1 expressed the highest levels of the costimulatory molecule CD80, suggesting that the migrant cDC1 were mature and activated for antigen presentation (Figure 2C).

**FIGURE 3:**
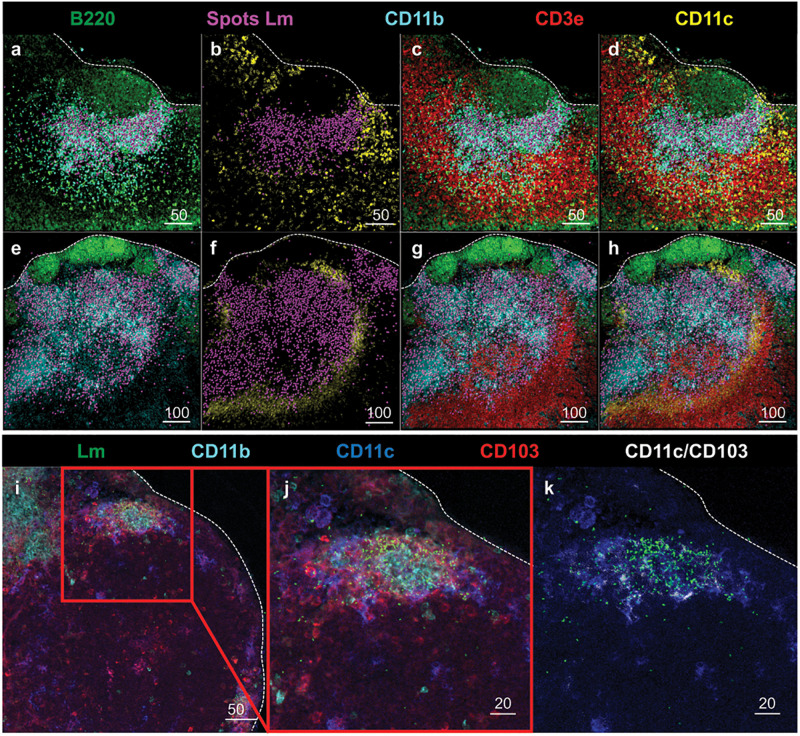
DC subsets surround regions of InlA^M^ *Lm* foci in the MLN after foodborne infection. **(a–h)** MLN from 2 Balb/c mice were harvested 2 days after foodborne InlA^M^ *Lm* infection. MLN cryosections were stained to identify B220 (green), InlA^M^ *Lm* (magenta), CD11b (cyan), CD3e (red) and CD11c (yellow). *Lm* staining was highlighted with magenta spheres using Imaris software. Scale bar represents 50 μm **(a–d)** and 100 μm **(e–h)**. **(i–k)** MLN from 2 Balb/c mice were harvested 3 days after foodborne InlA^M^ *Lm* infection. MLN cryosections were stained to identify InlA^M^ *Lm* (green), CD11b (cyan), CD11c (blue) and CD103 (red). Colocalization of CD11c and CD103 (CD11c/CD103) was highlighted in white using Imaris software. Scale bar represents 50 μm **(i)** and 20 μm **(j,k)**. All images are representative of at least three independent experiments, with 7–8 images taken for each timepoint.

To further evaluate the anatomical events surrounding intracellular trafficking of InlA^M^ *Lm* to the MLN, confocal microscopy provided additional context for the localization of InlA^M^ *Lm* with the early immune response in the MLN after foodborne InlA^M^ *Lm* infection. InlA^M^ *Lm* was localized to the deeper cortex in the T cell areas, where it was invariably associated with foci of CD11b^+^ CD11c^–^ cells (Figures 3a–h). Further assessment identified CD11c^+^ cells were associated with regions of InlA^M^ *Lm* foci (Figures 3a–h). InlA^M^ *Lm* visualized closer to the capsule and subcapsular sinus were surrounded by CD103^+^ and CD11c^+^ cells (Figures 3i–k). Thus, DC appear to be intricately involved in the early immune response to foodborne InlA^M^ *Lm* by surrounding foci of InlA^M^ *Lm* replication in tight association with T cells suggesting a potential site of antigen presentation.

### DC Play Diverse Roles in InlA^M^ *Lm* Dynamics After Foodborne Infection

**FIGURE 4:**
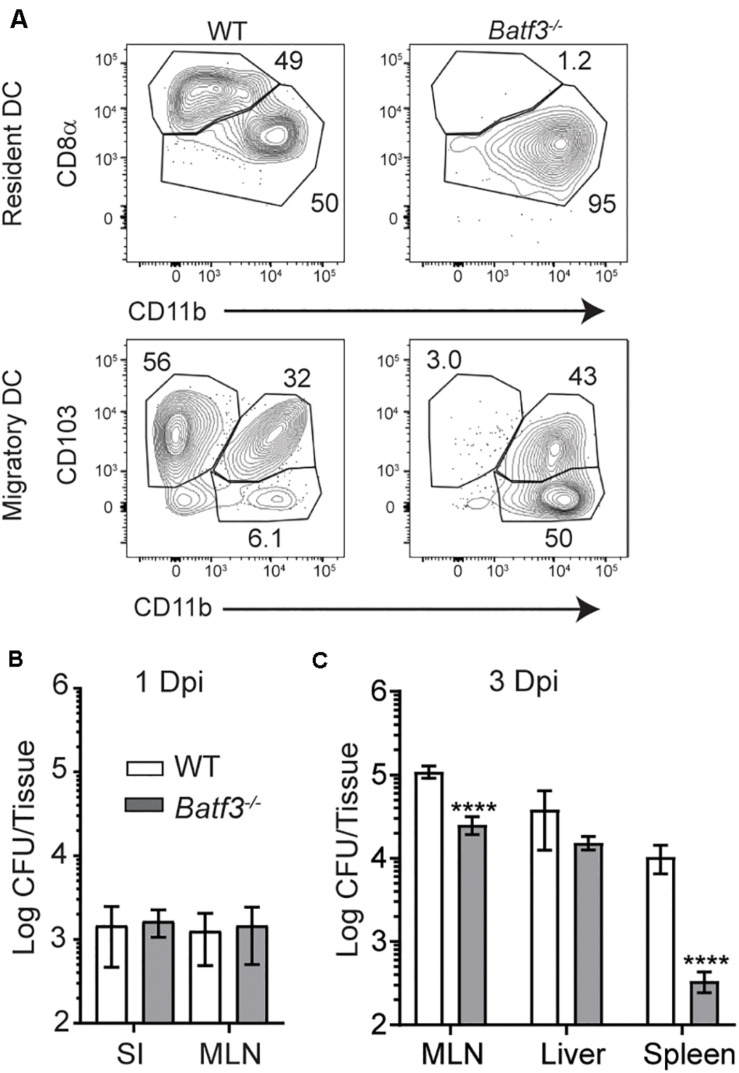
Defects in InlA^M^ *Lm* accumulation in the MLN after foodborne infection of *Batf3*^–/–^ mice. **(A)** WT and *Batf3*^–/–^ mice were infected with 1 × 10^10^ CFU InlA^M^ *Lm* 10403s. At 2 dpi, MLN cell suspensions were assessed by flow cytometry for DC. DC were identified as CD45^+^ CD19^–^ TCRβ^–^ F4/80^–^ Ly6C^–^ MHCII^hi^ CD11c^int^ single, live leukocytes (migratory DC) or CD45^+^ CD19^–^ TCRβ^–^ F4/80^–^ Ly6C^–^ MHCII^+^ CD11c^hi^ single, live leukocytes (resident DC). Resident DC subsets were identified based on their expression of CD8α and CD11b, while migratory DC were identified based on their expression of CD103 and CD11b. **(B,C)** WT and *Batf3*^–/–^ mice were infected with 2 × 10^9^ CFU InlA^M^ *Lm* 10403s. **(B,C)** The indicted tissues at 1 **(B)** or 3 **(C)** dpi were harvested, processed in saponin, and plated on BHI agar plates. Bacterial burdens were enumerated 24–48 h after plating. Data are cumulative of 2 experiments with *n* = 14–18 mice per group. Mann-Whitney tests were used to analyze the data: ****p < 0.0001.

After i.v. *Lm* infection, CD169^+^ macrophages in the spleen rapidly capture *Lm* and trans-infect splenic CD8α^+^ cDC1 (18). Splenic cDC1 have been implicated in the transport of *Lm* to their replicative niche in T cell zones and are critical for the establishment of productive infection (17, 18). As such, the role of cDC1 in the transit of InlA^M^ *Lm* and establishment of productive infection after foodborne infection was evaluated in *Batf3*^–/–^ mice, which lack resident CD8α^+^ and migrant CD103^+^ cDC1 (Figure 4A) (6, 17). The small intestine and MLN were assessed for replicating InlA^M^ *Lm* 1 day after foodborne infection of wild-type and *Batf3*^–/–^ Balb/c mice to assess colonization in the intestines and early dissemination to the MLN. While *Lm* burden is low in the spleen and liver of *Batf3*^–/–^ mice after i.v. infection (17), Batf3 deficiency did not reduce InlA^M^ *Lm* burden in the small intestine or MLN 1 day after infection (Figure 4B). This data indicates that early intestinal colonization and bacterial dissemination to the MLN after foodborne InlA^M^ *Lm* infection occurred in a Batf3-independent manner suggesting that cDC1 are not involved in these processes. However, by 3 dpi, *Batf3*^–/–^ MLN and spleens contained significantly less InlA^M^ *Lm* than WT MLN and spleens, suggesting that maximal bacterial accumulation in lymphoid tissues is Batf3-dependent (Figure 4C). Of note, the reduction observed in the MLN and spleen of *Batf3*^–/–^ mice after foodborne InlA^M^ *Lm* was much less substantial than that reported after i.v. infection (17). Moreover, lack of cDC1 did not diminish the InlA^M^ *Lm* burden from the liver. Collectively, these findings suggest that while cDC1 are dispensable for early intestinal colonization and dissemination to the MLN, they contribute to the maximal InlA^M^ *Lm* accumulation in lymphoid tissues.

**FIGURE 5:**
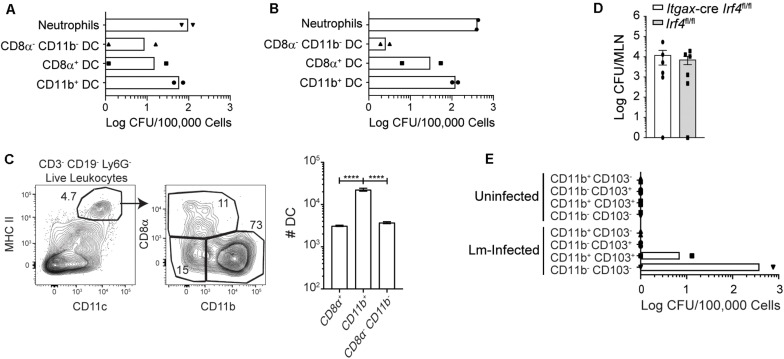
Most InlA^M^ *Lm* within DC subsets is associated with *Batf3*-independent DC. **(A,C)** Mice were infected with 2 × 10^9^ CFU InlA^M^ *Lm* 10403s. **(A)** At 3 dpi, MLN from 10 Balb/c mice were pooled together for cell sorting. Sorted subsets were plated on BHI agar containing streptomycin, and bacterial burdens were enumerated 24–48 h later. DC subsets were distinguished based on their expression of CD8α and CD11b. The graph depicts the mean and is cumulative of 2 independent experiments. Neutrophils were included as positive controls for both sets of experiments. **(B)** Balb/c mice were infected with 1 × 10^10^ CFU InlA^M^ *Lm* 10403s. At 3 dpi, 10 MLN were pooled together for cell sorting. Sorted DC subsets were plated onto BHI agar containing streptomycin, and burdens were enumerated 24–48 h after plating. The graph depicts the mean and is cumulative of 2 independent experiments. **(C)** MLN were harvested from Balb/c mice at 3 dpi, and dendritic cells subsets were analyzed via flow cytometry. Representative flow plots gated on B220^–^ TCRβ^–^ single, live leukocytes are shown. Graph depicts the quantification of dendritic cell subsets. **(D)** *Itgax*-cre *Irf4*^fl/fl^ and littermate (*Irf4*^fl/fl^) controls were infected with 2 × 10^9^ CFU InlA^M^ *Lm*-OVA 10403s. At 3 dpi, MLN were harvested, processed in saponin, and plated on BHI agar containing streptomycin. Bacterial burdens were enumerated 24–48 h after plating. Data are cumulative of 2 experiments. **(E)** Mesenteric lymphadenectomy was performed on B6 mice. After 4–6 weeks of recovery, mice were infected with 2 × 10^9^ CFU InlA^M^ *Lm* 10403s, and thoracic duct cannulation was performed at 1 dpi. Migratory DC subsets were sorted from the recovered lymph fluid, plated onto BHI agar plates containing streptomycin, and bacterial burdens were enumerated 24–48 h later. One-way ANOVA with Bonferroni’s multiple comparisons test was used to analyze data. ****p < 0.0001.

Based on the role of *Batf3* in bacterial accumulation in the MLN, the DC subsets carrying InlA^M^ *Lm* in the MLN were assessed at 3 dpi. CD8α^+^, CD11b^+^, and CD8α^–^ CD11b^–^ DC were sorted from pooled MLN of foodborne-infected WT mice, and bacterial burden was enumerated for each subset. A majority of the InlA^M^ *Lm* associated with sorted DC was found associated with the CD11b^+^ DC subset, both after foodborne infection with 2 × 10^9^ CFU and 1 × 10^10^ CFU (Figures 5A,B). Because CD11b^+^ DC encompass the largest proportion of the sorted subsets (Figure 5C), these findings imply that most of the bacteria associated with DC in the MLN are found within these cells. MLN-derived neutrophils and monocytes were also included in some experiments to serve as a positive control for the assay (Figure 5 and data not shown). Consistent with previous studies (22) these cells are associated with high numbers of InlA^M^ *Lm*. However, these cells are thought to be primarily recruited from the blood circulation (37–39). When bacterial burdens were assessed in mice lacking CD103^+^ CD11b^+^ DC, no differences were noted in the MLN 3 days after infection when compared to littermate controls (Figure 5D) (11, 12). This suggests that cDC2 are not required for bacterial accumulation in the MLN by 3 dpi. To assess whether CD11b^+^ cells were responsible for trafficking InlA^M^ *Lm* from the small intestines to the MLN, thoracic duct cannulation was used to determine whether DC subsets actively migrating in lymph fluid contained replicative InlA^M^ *Lm*. MLN were surgically removed from mice which were allowed to recover to redirect the flow of lymph fluid from the intestines to the thoracic duct. Lymphadenectomized mice received foodborne InlA^M^ *Lm*, and “pseudo-afferent” lymph fluid was collected from the thoracic duct over a period of ∼12 h 1 day after infection. Migratory DC subsets were sorted from the lymph, and bacterial burden was assessed for each DC subset (40). InlA^M^ *Lm* was detectable in both CD103^+^ CD11b^+^ and CD103^–^ CD11b^–^ DC early after foodborne infection (Figure 5E). Taken together, these data indicate that intestinal colonization and early accumulation in the MLN can occur in a Batf3-independent manner. Moreover, these findings suggest that multiple DC subsets may be able to transit *Lm* to the MLN.

### *Lm*-Specific CD8 T Cell Responses Are Greatly Reduced in *Batf3*^–/–^ Mice After Foodborne Infection

As InlA^M^ *Lm* infection was limited to gut tissues 1 day after foodborne infection, the location and duration of antigen presentation was analyzed by evaluating the proliferation of CFSE-labeled OT-I cells in different tissues after foodborne infection with InlA^M^ *Lm*-OVA. To assess major sites of antigen presentation after foodborne infection, CFSE-labeled CD45.1 OT-I T cells were transferred into CD45.2 B6 mice that had been infected with InlA^M^ *Lm*-OVA 1 day prior. MLN and spleens were harvested 16 h after adoptive transfer and cultured *in vitro* for 72 h. CFSE was diluted extensively in OT-I cells in the MLN but not in the spleen (Supplementary Figure S2A). This suggests that after foodborne InlA^M^ *Lm* infection, antigen presentation occurs early in the MLN. The duration of antigen presentation after foodborne infection was also assessed by transferring CFSE-labeled CD45.1 OT-I cells into foodborne-infected CD45.2 B6 mice at various times after infection (Supplementary Figure S2B). Donor cells were isolated 3 days after transfer and evaluated for CFSE dilution. Very few CFSE^+^ OT-I cells remained in the MLN or spleen after being transferred 3 days after foodborne infection, indicating that the cells underwent extensive proliferation. Transferring OT-I cells from day 6 after infection through day 14 resulted in a dramatic increase in the percentage of undiluted CFSE^+^ OT-I cells (Supplementary Figure S2B). These findings indicate that T cell proliferation declined over time and was undetectable by day 14 after infection. Thus, antigen was initially available to naïve T cells in mucosal tissues and was presented to T cells for 6–10 days after foodborne infection.

**FIGURE 6:**
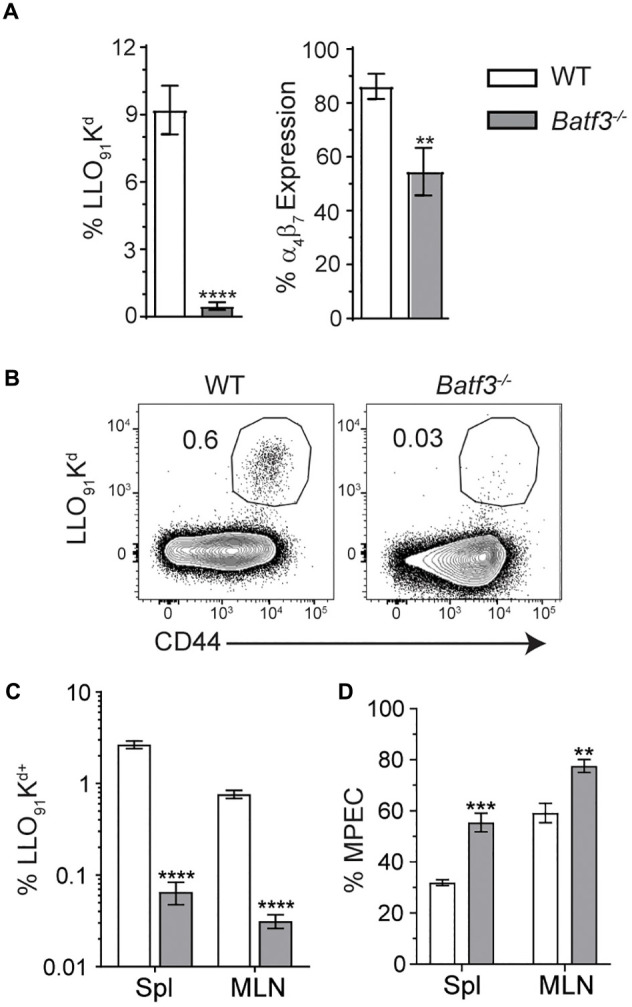
*Batf3*^–/–^ mice exhibit defects in InlA^M^ *Lm*-specific adaptive immune responses after foodborne infection. WT and *Batf3*^–/–^ mice were infected with 2 × 10^9^ CFU InlA^M^ *Lm* 10403s. **(A)** At 8 dpi, blood was collected from the tail veins of both groups of mice, and the samples were processed and stained for flow cytometry. LLO_91_-K^d^ tetramer staining was utilized to analyze InlA^M^ *Lm*-specific CD8 T cell responses, while α_4_β_7_ indicated gut homing potential. **(B–D)** At 9 dpi, spleens and MLN were analyzed by flow. LLO_91_-K^d^ tetramer staining indicated InlA^M^ *Lm*-specific CD8 T cells. MPEC were identified as KLRG-1^–^ CD127^+^ LLO_91_-K^d+^ CD8 T cells. **(B)** Representative tetramer staining is shown. Data are representative of at least 2 independent experiments with 7–8 mice per group. Unpaired *T*-tests were used to compare data sets: **p < 0.01; ***p < 0.001; ****p < 0.0001.

Batf3-dependent cDC1 drive the induction of cell-mediated immunity through their ability to cross-present cell-associated antigen to CD8 T cells (9, 10, 17). However, their role in the formation of adaptive immune responses after foodborne infection is not well understood and their requirement in the induction of CD8 T cell responses to *Lm* is not absolute after i.v. infection (17). Flow cytometric analyses of blood at 8 dpi yielded severely diminished yet detectable InlA^M^ *Lm*-specific CD8 T cell responses in *Batf3*^–/–^ mice compared to WT mice (Figure 6A). InlA^M^ *Lm*-specific CD8 T cells from *Batf3*^–/–^ mice also expressed lower levels of the integrin α_4_β_7_, suggesting that Batf3-dependent cDC1 also impart gut-homing capability (Figure 6A). This mirrors findings in *in vitro* studies which noted that CD103^+^ DC were required to drive effector CD8 T cell expression of the gut homing chemokine receptor CCR9 and integrin α_4_β_7_ (9). Further analysis of peak T cell responses at 9 dpi revealed significantly lower proportions of LLO_91_-K^d+^ CD8 T cells in both the MLN and spleens of *Batf3*^–/–^ mice (Figures 6B,C). Additionally, the differentiation of CD8 effector T cell populations was skewed toward memory potential in *Batf3*^–/–^ mice, which contained higher proportions of CD127^+^ KLRG-1^–^ memory precursor effector cells (MPEC) in both their MLN and spleens (Figure 6D). Meanwhile, an evaluation of InlA^M^ *Lm*-specific CD8 T cells responses at 9 dpi in the MLN of mice lacking migratory cDC2 in their intestinal LP and MLN (Supplementary Figure S3A) yielded no distinct differences when compared to littermate controls (Supplementary Figure S3B) (11, 12). Together, these findings suggest that cDC1, but not cDC2, are necessary to exert full, robust anti-InlA^M^ *Lm* adaptive immune responses after foodborne InlA^M^ *Lm* infection.

**FIGURE 7:**
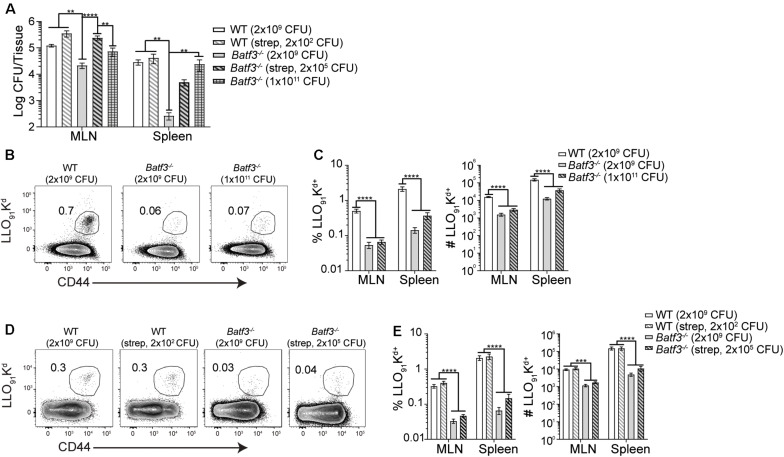
Induction of CD8 T cell responses are not rescued by restoring pathogen load. 20 mg of streptomycin was administered to the indicated groups of WT and *Batf3*^–/–^ Balb/c mice 1 day prior to infection. Mice were infected with the indicated doses of InlA^M^ *Lm* 10403s. **(A)** At 3 dpi, MLN and spleens were harvested, processed, and plated on BHI agar to enumerate InlA^M^ *Lm* burden. Data are pooled from 5 to 6 individual experiments and included both male and female mice (*n* = 7–41). **(B–E)** At 9 dpi, MLN and spleens were harvested and processed for flow cytometry. LLO_91_-K^d^ tetramer staining was utilized to analyze InlA^M^ *Lm*-specific CD8 T cell responses. Data are pooled from 4 to 6 individual experiments and included both male and female mice (*n* = 12–20 mice per group). **(B,D)** Representative tetramer staining is shown. Bacterial burdens were compared using one-way ANOVA with Kruskal-Wallis non-parametric test. Cell populations were compared using one-way ANOVA with Bonferroni’s multiple comparisons test: **p < 0.01; ***p < 0.001; ****p < 0.0001.

After i.v. *Lm* infection, defects in *Lm*-specific CD8 T cell responses in *Batf3*^–/–^ mice were observed. However, when the infection dose administered to *Batf3*^–/–^ mice was increased, bacterial burden and *Lm*-specific CD8 T cell responses were restored suggesting that the defect in the CD8 T cell response was due to diminished bacterial burdens in *Batf3*^–/–^ mice (17). Therefore, whether restoration of pathogen burden could be achieved in *Batf3*^–/–^ mice after foodborne infection was determined with two distinct approaches. One cohort of *Batf3*^–/–^ mice received an infection dose of 1 × 10^11^ CFU while another cohort of *Batf3*^–/–^ were treated with 20 mg streptomycin by gavage prior to infection with 2 × 10^5^ CFU InlA^M^ *Lm*. Treatment with streptomycin prior to infection eliminates colonization resistance provided by the normal intestinal microbiota and promotes intestinal invasion and dissemination after low dose foodborne *Lm* infection (41). Both approaches to restore pathogen burden in the MLN and spleen of *Batf3*^–/–^ mice after foodborne infection yielded similar levels of InlA^M^ *Lm* in the MLN and spleen as control mice (Figure 7A). Thus, CD8 T cell responses could be evaluated in WT and *Batf3*^–/–^ mice with similar pathogen loads. Despite their ability to restore pathogen burden, neither high-dose infection nor pretreatment with streptomycin were capable of rescuing InlA^M^ *Lm*-specific CD8 T cell responses in the MLN or spleen of *Batf3*^–/–^ mice (Figures 7B–E). This inability to rescue InlA^M^ *Lm*-specific CD8 T cell responses was not restricted to lymphoid tissues, as intestinal intraepithelial lymphocyte (IEL) responses were also diminished using both methods of pathogen burden restoration (Supplementary Figure S4). These findings demonstrate that *Batf3* is critical for the induction of InlA^M^ *Lm*-specific CD8 T cell responses after foodborne infection, and that restoration of pathogen burden in *Batf3*^–/–^ mice is unable to rescue the CD8 T cell defect. This is in contrast to the restoration of pathogen burden during i.v. *Lm* infection, where *Lm*-specific CD8 T cell responses were rescued in *Batf3*^–/–^ mice (16, 17). Altogether, these data suggest that cDC1 may play an important role in driving effector CD8 T cell responses after foodborne InlA^M^ *Lm* infection.

## Discussion

Route of infection and pathogen dissemination from the site of infection play an important role in the generation of anti-pathogen immune responses. A physiologic infection method that more closely resembles human exposure to foodborne pathogens and oral vaccines to assess early immune events that impact dissemination of the bacterial pathogen InlA^M^ *Lm* and induction of T cell immunity was utilized. After foodborne infection, InlA^M^ *Lm* appears restricted to the gut tissues before disseminating to extraintestinal sites like the liver and spleen. Balb/c and B6 mice infected with 2 × 10^9^ CFU of InlA^M^ *Lm* did not contain detectable replicating bacteria in the liver or spleen until 2 days after foodborne infection. While foodborne infection of Balb/cBy (a substrain of Balb/c) mice results in an extracellular lifecycle phase in dissemination to or replication within the MLN (21), foodborne infection of Balb/c mice yielded predominately intracellular InlA^M^ *Lm* in the MLN as gentamicin treatment or depletion of neutrophils did not reduce the bacterial burden. Balb/cBy mice are known to be highly susceptible to InlA^M^ *Lm* infection (42, 43), and foodborne infection with 2 × 10^9^ CFU of InlA^M^ *Lm* resulted in substantially higher bacterial burdens in Balb/cBy mice that was associated with reduced survival. The presence of extracellular InlA^M^ *Lm* replication may contribute to the enhanced susceptibility of Balb/cBy mice or be a consequence of higher bacterial burdens associated with enhanced susceptibility. Regardless, both models of foodborne infection found that InlA^M^ *Lm* was largely extracellular in the liver, which is also consistent with what has been reported after i.v. *Lm* infection (44).

The implication that InlA^M^ *Lm* is primarily intracellular within the MLN led to the hypothesis that InlA^M^ *Lm* is trafficked via immune cells migrating from the gut lamina propria. As DC represent the major subset of lymph migrant immune cells from the gut, DC subsets responding to foodborne InlA^M^ *Lm* infection were assessed. Flow cytometric analysis after foodborne InlA^M^ *Lm* infection showed that migratory DC accumulated in the MLN by 3 dpi, while confocal microscopy revealed that InlA^M^ *Lm* in the MLN was mostly contained within foci in close proximity to CD11b^+^ and CD11c^+^ cells (some of which also expressed CD103). Additionally, splenic cDC1 are known to shuttle *Lm* from CD169^+^ macrophages to the T cell zones after i.v. infection (17, 18). Assessment of bacterial burden in *Batf3*^–/–^ and wild-type mice demonstrated comparable InlA^M^ *Lm* burden in the small intestine and MLN at 1 dpi suggesting that cDC1 are dispensable for initial intestinal colonization and early accumulation in the MLN. However, reductions in the MLN and spleen were observable by 3 dpi, suggesting that cDC1 are needed for maximal accumulation of foodborne InlA^M^ *Lm*. Interestingly, there were no significant differences in bacterial burden in the livers of *Batf3*^–/–^ and WT mice, suggesting that cDC1 are dispensable for hepatic bacterial accumulation after foodborne InlA^M^ *Lm* infection and this is consistent with the large reservoir of extracellular replication observed in that tissue. Of note, this is in contrast with i.v. *Lm* infection of *Batf3*^–/–^ mice, in which the inability to establish infection is observed in the spleen and liver suggesting that route of infection impacts the role of cDC1 in potentiating InlA^M^ *Lm* infection (17).

Due to the finding that *Batf3*^–/–^ mice display defects in maximal bacterial accumulation in the MLN after foodborne InlA^M^ *Lm* infection, the DC subsets which contain InlA^M^ *Lm* in the MLN after foodborne infection were determined. CD11b^+^ DC contained the majority of the DC-associated InlA^M^ *Lm* found in the MLN at 3 dpi and represented the largest DC subset at this time. InlA^M^ *Lm* was also found within pseudo-afferent lymph CD11b^+^ CD103^+^ and CD103^–^ CD11b^–^ DC 1 day after foodborne InlA^M^ *Lm* infection. CD103^–^ CD11b^–^ DC migrate in intestinal lymph and are capable of presenting antigen to both CD4 and CD8 T cells (14). Thus, replicative InlA^M^ *Lm* can transit from the gut to the MLN in association with CD11b^+^ CD103^+^ and CD103^–^ CD11b^–^ DC subsets consistent with the findings that suggest early intestinal colonization and accumulation in the MLN is able to occur independently of cDC1. As bacterial burdens in *Itgax*-cre *Irf4*^fl/fl^ mice, which lack CD103^+^ CD11b^+^ DC, were comparable to those of their littermate controls at 3 dpi, this subset does not appear to be required for *Lm* accumulation or control within the MLN. Gentamicin treatment did not reduce bacterial burden in the MLN when administered at 4 hpi, suggesting that DC from pseudo-afferent lymph harbored intracellular replicative InlA^M^ *Lm* during transit to the MLN. Collectively, these data suggest that while cDC2 may be capable of carrying *Lm* to the MLN, neither cDC1 nor cDC2 are required for the initial dissemination of InlA^M^ *Lm* from the gut to the MLN. Whether redundancies allow cDC2 to perform this function in the absence of cDC1 and vice versa is unclear and limitations with the thoracic duct cannulation procedure and the sensitivity of the assay preclude eliminating other cells as a source of initial transit. However, collectively, these findings reinforce the idea that diverse DC subsets may be involved in bacterial transit following foodborne infection. Despite the finding that InlA^M^ *Lm* is most prevalent among cDC2 in the MLN, it is well-established that cDC1 play a critical role in the generation of CD8 T cell responses due to their ability to cross-present cell-associated antigens (19, 45–47). Therefore, these findings suggest that diverse DC subsets may traffic InlA^M^ *Lm* to the MLN, where cDC1 process and present antigens to naïve CD8 T cells, resulting in robust adaptive immune responses. Thus, these findings are consistent with the notion of a tight bottleneck during invasion of the gastrointestinal tract (48) and suggest that diverse DC subsets dynamically associate with InlA^M^ *Lm* early after foodborne infection in order to disseminate the pathogen and induce CD8 T cell responses.

Foodborne infection of Balb/cBy mice demonstrated that a large proportion of InlA^M^ *Lm* adhered to Ly6C^hi^ monocytes (22). However, most InlA^M^ *Lm* was contained intracellularly after foodborne infection of Balb/c mice in this study and most monocytes in the MLN are likely derived from the blood, the relevance of this population *in vivo* was not assessed. It is well documented that monocytes are critical for control of *Lm* (49) and further studies might address differences in this important cell type to pathogen resistance in Balb/c or B6 mice and susceptibility in Balb/cBy mice. Furthermore, intestinal CX3CR1^+^ mononuclear phagocytes act as vessels to traffic *Salmonella enterica* from the intestines to the MLN (50). However, whether these cells more closely align with macrophages or dendritic cells is open to interpretation (51). Finally, loss of CD169^+^ macrophages did not impact bacterial burden in the MLN or small intestines after foodborne InlA^M^ *Lm* infection (18). This suggests that while these marginal zone macrophages play an important role filtering the blood after i.v. infection, they do not play a similar role in filtering intestinal lymph containing cell-autonomous InlA^M^ *Lm* after foodborne infection.

These findings suggest that cDC1 play an important role in the generation of robust, protective InlA^M^ *Lm*-specific CD8 T cell responses after foodborne infection. Numerous publications have outlined the importance of cDC1 in generating CD8 T cell responses to intracellular pathogens or apoptotic cell-associated antigens (19, 45–47, 52). A recent study has also demonstrated that *Batf3* is dispensable for InlA^M^ *Lm*-specific effector CD8 T cells after foodborne infection (53), suggesting the validity of our approach to assess cDC1 in effector CD8 T cell responses. This study revealed that *Batf3*^–/–^ mice displayed distinct defects in both effector T cell accumulation and α_4_β_7_ expression. Expression of α_4_β_7_ is imprinted on T cells by DC, and allows for the migration of lymphocytes to the intestines via interactions with its ligand, MAdCAM-1 (54). Furthermore, the few antigen-specific CD8 T cells present in *Batf3*^–/–^ mice were skewed toward MPEC differentiation. Altogether, these findings indicate that *Batf3* is needed to generate these responses and suggest cDC1 are needed for the induction of effector CD8 T cells after foodborne InlA^M^ *Lm* infection. Previous studies have noted that the defects in T cell responses in *Batf3*^–/–^ mice after i.v. *Lm* infection were attributable to low pathogen burden. Subsequent restoration of pathogen burden through increased i.v. infection dose was able to rescue *Lm*-specific CD8 T cell responses in *Batf3*^–/–^ mice, suggesting that other antigen presenting cells are capable of eliciting a CD8 T cell response to *Lm* infection (17). As methods used to normalize bacterial burden were unable to restore the effector CD8 T cell response after foodborne infection, *Batf3* is playing a critical role in the development of effector CD8 T cells after foodborne infection. Additionally, the heterogeneity of splenic and MLN DC subsets could impact the differences noted between foodborne and i.v. infection models after pathogen burden restoration. Splenic DC are comprised of only lymphoid-resident DC, while both migratory and lymphoid-resident DC are present in the MLN. While splenic cDC2 in *Batf3*^–/–^ mice may be more poised to present antigen upon sufficient pathogen burden after i.v. infection, differences in the composition and antigen acquisition, processing and presentation capabilities within the *Batf3*^–/–^ MLN could limit the ability to drive CD8 T cell responses after foodborne infection. Previous studies of foodborne infection found that once inside cDC, GFP-expressing InlA^M^ *Lm* was not able to efficiently escape the vacuole and gain access to the cytosol (23). This inability to successfully enter the cytosol could limit the potential of cDC to process and present antigen. In this context, cDC2, which are poor at cross-presenting antigens *in vivo* (55) might be inefficient at inducing a CD8 T cell response. The inability to cross-present antigen *in vivo* was further reflected in the finding that mice lacking cDC2 did not display defects in InlA^M^ *Lm*-specific CD8 T cell responses compared to littermate controls. Additionally, methods of pathogen burden restoration could also result in larger proportions of extracellular bacteria in the *Batf3*^–/–^ mice, which may be shuttled to the MLN via other cell types or cell-autonomously. Although more studies will need to be performed to examine this, these potential alternate methods of transit could impact antigen uptake and subsequently, presentation to naïve T cells. Finally, while previous studies have demonstrated a profound *Batf3* cell-extrinsic effect on the induction of *Lm*-specific CD8 T cell responses (16, 17), mice used in this study also lack *Batf3* expression in CD8 T cells that may influence the T cell responses at higher infectious doses.

Collectively, this study has demonstrated that DC play diverse and dynamic roles in early responses to foodborne InlA^M^ *Lm* and suggested that cDC1 are needed for the induction of InlA^M^ *Lm*-specific effector CD8 T cell response. Recent works have demonstrated that vaccines stimulating cDC1 can induce effective anti-tumor CD8 T cell responses and that cDC1 are critical for the formation of robust tissue-resident memory T (T_RM_) cell responses in the skin after vaccinia virus infection, making these DC potential targets for future vaccine development (56, 57). However, other studies have now implicated IRF4-dependent cDC2 in lung T_RM_ formation after influenza infection (58). Therefore, understanding mucosal DC is critical to learning more about their influence on the formation of effector and memory T cell populations, and how the generation of these T cell populations can be utilized for the development of potent vaccines.

## Data Availability Statement

The raw data supporting the conclusions of this article will be made available by the authors, without undue reservation.

## Ethics Statement

The animal study was reviewed and approved by Institutional Animal Care and Use Committee’s at Stony Brook University and UConn Health and the Animal Welfare Review Board and UK Home Office for cannulations performed at the University of Glasgow.

## Author Contributions

JI, DX, PR, ZQ, PP, CK, AA, AB-B, and Q-MP performed the experiments. SM, LL, KK, LP, and BS contributed to the study design. JI and BS designed the experiments, analyzed and interpreted the data, and wrote the manuscript. All authors contributed to the article and approved the submitted version.

## Conflict of Interest

The authors declare that the research was conducted in the absence of any commercial or financial relationships that could be construed as a potential conflict of interest.

## References

[1] Zhu Q Gooneratne R Hussain MA. *Listeria monocytogenes* in fresh produce: outbreaks, prevalence and contamination levels. *Foods.* (2017) 6:21. 10.3390/foods6030021 28282938 PMC5368540

[2] Salama PJ Embarek PKB Bagaria J Fall IS. Learning from *Listeria*: safer food for all. *Lancet.* (2018) 391:2305–6. 10.1016/S0140-6736(18)31206-6 29900862

[3] Wood LM Paterson Y. Attenuated *Listeria monocytogenes*: a powerful and versatile vector for the future of tumor immunotherapy. *Front Cell Infect Microbiol.* (2014) 4:51. 10.3389/fcimb.2014.00051 24860789 PMC4026700

[4] Banchereau J Briere F Caux C Davoust J Lebecque S Liu YJ Immunobiology of dendritic cells. *Annu Rev Immunol.* (2000) 18:767–811. 10.1146/annurev.immunol.18.1.767 10837075

[5] Merad M Sathe P Helft J Miller J Mortha A. The dendritic cell lineage: ontogeny and function of dendritic cells and their subsets in the steady state and the inflamed setting. *Annu Rev Immunol.* (2013) 31:563–604. 10.1146/annurev-immunol-020711-074950 23516985 PMC3853342

[6] Edelson BT Kc W Juang R Kohyama M Benoit LA Klekotka PA Peripheral CD103+ dendritic cells form a unified subset developmentally related to CD8alpha+ conventional dendritic cells. *J Exp Med.* (2010) 207:823–36. 10.1084/jem.20091627 20351058 PMC2856032

[7] Persson EK Scott CL Mowat AM Agace WW. Dendritic cell subsets in the intestinal lamina propria: ontogeny and function. *Eur J Immunol.* (2013) 43:3098–107. 10.1002/eji.201343740 23966272 PMC3933733

[8] Guilliams M Ginhoux F Jakubzick C Naik SH Onai N Schraml BU Dendritic cells, monocytes and macrophages: a unified nomenclature based on ontogeny. *Nat Rev Immunol.* (2014) 14:571–8. 10.1038/nri3712 25033907 PMC4638219

[9] Johansson-Lindbom B Svensson M Pabst O Palmqvist C Marquez G Forster R Functional specialization of gut CD103+ dendritic cells in the regulation of tissue-selective T cell homing. *J Exp Med.* (2005) 202:1063–73. 10.1084/jem.20051100 16216890 PMC2213212

[10] Johansson-Lindbom B Svensson M Wurbel MA Malissen B Marquez G Agace W. Selective generation of gut tropic T cells in gut-associated lymphoid tissue (GALT): requirement for GALT dendritic cells and adjuvant. *J Exp Med.* (2003) 198:963–9. 10.1084/jem.20031244 12963696 PMC2194196

[11] Persson EK Uronen-Hansson H Semmrich M Rivollier A Hagerbrand K Marsal J IRF4 transcription-factor-dependent CD103(+)CD11b(+) dendritic cells drive mucosal T helper 17 cell differentiation. *Immunity.* (2013) 38:958–69. 10.1016/j.immuni.2013.03.009 23664832

[12] Schlitzer A McGovern N Teo P Zelante T Atarashi K Low D IRF4 transcription factor-dependent CD11b+ dendritic cells in human and mouse control mucosal IL-17 cytokine responses. *Immunity.* (2013) 38:970–83. 10.1016/j.immuni.2013.04.011 23706669 PMC3666057

[13] Bar-On L Birnberg T Lewis KL Edelson BT Bruder D Hildner K CX3CR1+ CD8alpha+ dendritic cells are a steady-state population related to plasmacytoid dendritic cells. *Proc Natl Acad Sci USA.* (2010) 107:14745–50. 10.1073/pnas.1001562107 20679228 PMC2930429

[14] Cerovic V Houston SA Scott CL Aumeunier A Yrlid U Mowat AM Intestinal CD103(-) dendritic cells migrate in lymph and prime effector T cells. *Mucosal Immunol.* (2013) 6:104–13. 10.1038/mi.2012.53 22718260

[15] Williams MA Schmidt RL Lenz LL. Early events regulating immunity and pathogenesis during *Listeria monocytogenes* infection. *Trends Immunol.* (2012) 33:488–95. 10.1016/j.it.2012.04.007 22677184 PMC3440530

[16] Liu D Yin X Olyha SJ Nascimento MSL Chen P White T IL-10-dependent crosstalk between murine marginal zone B cells, macrophages, and CD8alpha(+) dendritic cells promotes *Listeria monocytogenes* infection. *Immunity.* (2019) 51:64–76.e7. 10.1016/j.immuni.2019.05.011 31231033 PMC6685086

[17] Edelson BT Bradstreet TR Hildner K Carrero JA Frederick KE Kc W CD8alpha(+) dendritic cells are an obligate cellular entry point for productive infection by *Listeria monocytogenes*. *Immunity.* (2011) 35:236–48. 10.1016/j.immuni.2011.06.012 21867927 PMC3172670

[18] Perez OA Yeung ST Vera-Licona P Romagnoli PA Samji T Ural BB CD169(+) macrophages orchestrate innate immune responses by regulating bacterial localization in the spleen. *Sci Immunol.* (2017) 2:eaah5520. 10.1126/sciimmunol.aah5520 28986418 PMC5969998

[19] Hildner K Edelson BT Purtha WE Diamond M Matsushita H Kohyama M Batf3 deficiency reveals a critical role for CD8alpha+ dendritic cells in cytotoxic T cell immunity. *Science.* (2008) 322:1097–100. 10.1126/science.1164206 19008445 PMC2756611

[20] Qiu Z Khairallah C Sheridan BS. *Listeria monocytogenes*: a model pathogen continues to refine our knowledge of the CD8 T cell response. *Pathogens.* (2018) 7:55. 10.3390/pathogens7020055 29914156 PMC6027175

[21] Jones GS Bussell KM Myers-Morales T Fieldhouse AM Bou Ghanem EN D’Orazio SE. Intracellular *Listeria monocytogenes* comprises a minimal but vital fraction of the intestinal burden following foodborne infection. *Infect Immun.* (2015) 83:3146–56. 10.1128/IAI.00503-15 26015479 PMC4496611

[22] Jones GS D’Orazio SE. Monocytes are the predominant cell type associated with *Listeria monocytogenes* in the Gut, but they do not serve as an intracellular growth niche. *J Immunol.* (2017) 198:2796–804. 10.4049/jimmunol.1602076 28213502 PMC5360488

[23] Jones GS Smith VC D’Orazio SEF. *Listeria monocytogenes* replicate in bone marrow-derived CD11c(+) cells but not in dendritic cells isolated from the murine gastrointestinal tract. *J Immunol.* (2017) 199:3789–97. 10.4049/jimmunol.1700970 29055001 PMC5698106

[24] Bonazzi M Lecuit M Cossart P. *Listeria monocytogenes* internalin and E-cadherin: from bench to bedside. *Cold Spring Harb Perspect Biol.* (2009) 1:a003087. 10.1101/cshperspect.a003087 20066101 PMC2773623

[25] Sheridan BS Lefrancois L. Regional and mucosal memory T cells. *Nat Immunol.* (2011) 12:485–91. 21739671 10.1038/ni.2029 PMC3224372

[26] Wollert T Pasche B Rochon M Deppenmeier S van den Heuvel J Gruber AD Extending the host range of *Listeria monocytogenes* by rational protein design. *Cell.* (2007) 129:891–902. 10.1016/j.cell.2007.03.049 17540170

[27] Milling S Yrlid U Cerovic V MacPherson G. Subsets of migrating intestinal dendritic cells. *Immunol Rev.* (2010) 234:259–67. 10.1111/j.0105-2896.2009.00866.x 20193024

[28] Milling SW Jenkins C MacPherson G. Collection of lymph-borne dendritic cells in the rat. *Nat Protoc.* (2006) 1:2263–70. 10.1038/nprot.2006.315 17406466

[29] Qiu Z Sheridan BS. Isolating lymphocytes from the mouse small intestinal immune system. *J Vis Exp.* (2018) 132:57281. 10.3791/57281 29553537 PMC5931411

[30] Sheridan BS Lefrancois L. Isolation of mouse lymphocytes from small intestine tissues. *Curr Protoc Immunol.* (2012) Chapter 3:Unit3.19. 10.1002/0471142735.im0319s99 23129154 PMC8128104

[31] Turner DL Cauley LS Khanna KM Lefrancois L. Persistent antigen presentation after acute vesicular stomatitis virus infection. *J Virol.* (2007) 81:2039–46. 10.1128/JVI.02167-06 17151119 PMC1797569

[32] Zammit DJ Turner DL Klonowski KD Lefrancois L Cauley LS. Residual antigen presentation after influenza virus infection affects CD8 T cell activation and migration. *Immunity.* (2006) 24:439–49. 10.1016/j.immuni.2006.01.015 16618602 PMC2861289

[33] Edson RS Terrell CL. The aminoglycosides: streptomycin, kanamycin, gentamicin, tobramycin, amikacin, netilmicin, and sisomicin. *Mayo Clin Proc.* (1987) 62:916–20. 10.1016/s0025-6196(12)65048-4 3657309

[34] Witter AR Okunnu BM Berg RE. The essential role of neutrophils during infection with the intracellular bacterial pathogen *Listeria monocytogenes*. *J Immunol.* (2016) 197:1557–65. 10.4049/jimmunol.1600599 27543669 PMC4995063

[35] Aoshi T Carrero JA Konjufca V Koide Y Unanue ER Miller MJ. The cellular niche of *Listeria monocytogenes* infection changes rapidly in the spleen. *Eur J Immunol.* (2009) 39:417–25. 10.1002/eji.200838718 19130474 PMC2749683

[36] Cerovic V Bain CC Mowat AM Milling SW. Intestinal macrophages and dendritic cells: what’s the difference? *Trends Immunol.* (2014) 35:270–7. 10.1016/j.it.2014.04.003 24794393

[37] Geissmann F Jung S Littman DR. Blood monocytes consist of two principal subsets with distinct migratory properties. *Immunity.* (2003) 19:71–82. 10.1016/s1074-7613(03)00174-2 12871640

[38] Serbina NV Jia T Hohl TM Pamer EG. Monocyte-mediated defense against microbial pathogens. *Annu Rev Immunol.* (2008) 26:421–52. 10.1146/annurev.immunol.26.021607.090326 18303997 PMC2921669

[39] Shi C Pamer EG. Monocyte recruitment during infection and inflammation. *Nat Rev Immunol.* (2011) 11:762–74. 10.1038/nri3070 21984070 PMC3947780

[40] Scott CL Wright PB Milling SW Mowat AM. Isolation and identification of conventional dendritic cell subsets from the intestine of mice and men. *Methods Mol Biol.* (2016) 1423:101–18. 10.1007/978-1-4939-3606-9_7 27142011

[41] Becattini S Littmann ER Carter RA Kim SG Morjaria SM Ling L Commensal microbes provide first line defense against *Listeria monocytogenes* infection. *J Exp Med.* (2017) 214:1973–89. 10.1084/jem.20170495 28588016 PMC5502438

[42] Cheers C McKenzie IF. Resistance and susceptibility of mice to bacterial infection: genetics of listeriosis. *Infect Immun.* (1978) 19:755–62. 305895 10.1128/iai.19.3.755-762.1978 PMC422253

[43] Cheers C McKenzie IF Pavlov H Waid C York J. Resistance and susceptibility of mice to bacterial infection: course of listeriosis in resistant or susceptible mice. *Infect Immun.* (1978) 19: 763–70. 417029 10.1128/iai.19.3.763-770.1978 PMC422254

[44] Conlan JW. Early pathogenesis of *Listeria monocytogenes* infection in the mouse spleen. *J Med Microbiol.* (1996) 44:295–302. 10.1099/00222615-44-4-295 8606358

[45] Desai P Tahiliani V Abboud G Stanfield J Salek-Ardakani S. Batf3-dependent dendritic cells promote optimal CD8 T cell responses against respiratory poxvirus infection. *J Virol.* (2018) 92:JVI.00495-18. 10.1128/JVI.00495-18 29875235 PMC6069197

[46] Desch AN Randolph GJ Murphy K Gautier EL Kedl RM Lahoud MH CD103+ pulmonary dendritic cells preferentially acquire and present apoptotic cell-associated antigen. *J Exp Med.* (2011) 208:1789–97. 10.1084/jem.20110538 21859845 PMC3171085

[47] Torti N Walton SM Murphy KM Oxenius A. Batf3 transcription factor-dependent DC subsets in murine CMV infection: differential impact on T-cell priming and memory inflation. *Eur J Immunol.* (2011) 41:2612–8. 10.1002/eji.201041075 21604258

[48] Zhang T Abel S Abel Zur Wiesch P Sasabe J Davis BM Higgins DE Deciphering the landscape of host barriers to *Listeria monocytogenes* infection. *Proc Natl Acad Sci USA.* (2017) 114:6334–9. 10.1073/pnas.1702077114 28559314 PMC5474794

[49] Serbina NV Salazar-Mather TP Biron CA Kuziel WA Pamer EG. TNF/iNOS-producing dendritic cells mediate innate immune defense against bacterial infection. *Immunity.* (2003) 19:59–70. 10.1016/s1074-7613(03)00171-7 12871639

[50] Diehl GE Longman RS Zhang JX Breart B Galan C Cuesta A Microbiota restricts trafficking of bacteria to mesenteric lymph nodes by CX(3)CR1(hi) cells. *Nature.* (2013) 494:116–20. 10.1038/nature11809 23334413 PMC3711636

[51] Bravo-Blas A Utriainen L Clay SL Kastele V Cerovic V Cunningham AF *Salmonella enterica* serovar typhimurium travels to mesenteric lymph nodes both with host cells and autonomously. *J Immunol.* (2019) 202:260–7. 10.4049/jimmunol.1701254 30487173 PMC6305795

[52] Mashayekhi M Sandau MM Dunay IR Frickel EM Khan A Goldszmid RS CD8alpha(+) dendritic cells are the critical source of interleukin-12 that controls acute infection by *Toxoplasma gondii* tachyzoites. *Immunity.* (2011) 35:249–59. 10.1016/j.immuni.2011.08.008 21867928 PMC3171793

[53] Qiu Z Khairallah C Romanov G Sheridan BS. Cutting edge: Batf3 expression by CD8 T cells critically regulates the development of memory populations. *J Immunol.* (2020) 205:901–6. 10.4049/jimmunol.2000228 32669309 PMC7539233

[54] Mora JR Bono MR Manjunath N Weninger W Cavanagh LL Rosemblatt M Selective imprinting of gut-homing T cells by Peyer’s patch dendritic cells. *Nature.* (2003) 424:88–93. 10.1038/nature01726 12840763

[55] den Haan JM Lehar SM Bevan MJ. CD8(+) but not CD8(-) dendritic cells cross-prime cytotoxic T cells in vivo. *J Exp Med.* (2000) 192:1685–96. 11120766 10.1084/jem.192.12.1685 PMC2213493

[56] Hammerich L Marron TU Upadhyay R Svensson-Arvelund J Dhainaut M Hussein S Systemic clinical tumor regressions and potentiation of PD1 blockade with in situ vaccination. *Nat Med.* (2019) 25:814–24. 10.1038/s41591-019-0410-x 30962585

[57] Iborra S Martinez-Lopez M Khouili SC Enamorado M Cueto FJ Conde-Garrosa R Optimal generation of tissue-resident but not circulating memory T cells during viral infection requires crosspriming by DNGR-1(+) dendritic cells. *Immunity.* (2016) 45:847–60. 10.1016/j.immuni.2016.08.019 27692611 PMC5074364

[58] Ainsua-Enrich E Hatipoglu I Kadel S Turner S Paul J Singh S IRF4-dependent dendritic cells regulate CD8(+) T-cell differentiation and memory responses in influenza infection. *Mucosal Immunol.* (2019) 12:1025–37. 10.1038/s41385-019-0173-1 31089186 PMC6527354

