# Supplementary material for: Mucosal CD8 T Cell Responses Are Shaped by Batf3-DC After Foodborne Listeria monocytogenes Infection
Source: Front Immunol. 2020 Sep 11;11:575967. doi: 10.3389/fimmu.2020.575967 (PMC7518468; doi:10.3389/fimmu.2020.575967)
Supplement: Supplementary file 1 [file Presentation_1.pdf]

Supplementary Material

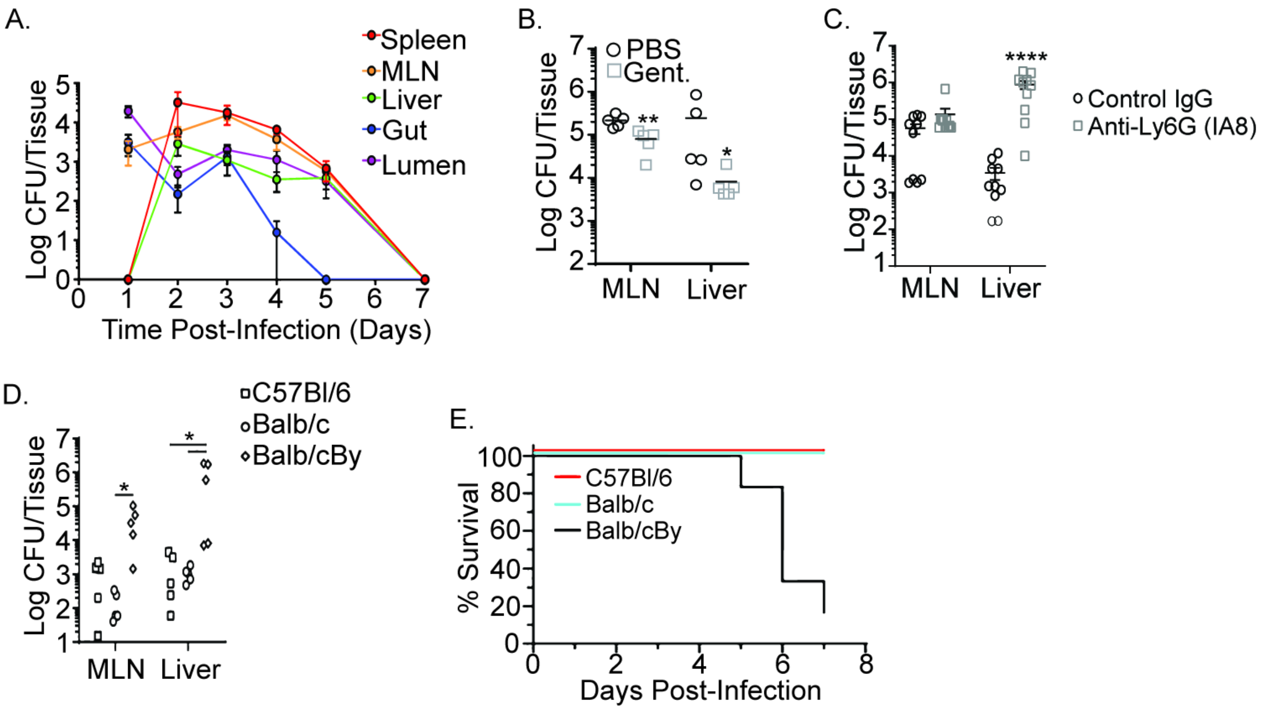

Figure S1. *Lm* kinetics in B6, Balb/c, and Balb/cBy mice after foodborne infection. (**A**) B6 mice were foodborne infected with 2x10^9 CFU InlA^M *Lm*-OVA 10403s. At the indicated time points, tissues were harvested, processed with saponin, and plated onto BHI agar containing streptomycin. Bacterial burdens were enumerated 24-48 hours after plating. (**A**) 1 and 3 dpi burdens have been repeated at least 3 times. The complete time course has been performed 1 time in B6 mice and 1 time in Balb/c mice (**Figure 1**). For this experiment, tissues were harvested from 5 mice at each timepoint. (**B**) Mice were foodborne infected with 2x10^{10} CFU InlA^M r*Lm* and were treated with either gentamicin or PBS at 4 hpi. At 3 dpi, MLN and livers were harvested, and bacterial burdens were quantified. (**C**) Balb/c mice were foodborne infected with 2x10^9 CFU InlA^M *Lm* 10403s and treated with 250ug anti-Ly6G antibody (clone IA8) or control IgG via intraperitoneal injection at days -1, 0, 1, and 2. At 3dpi, MLN and livers were harvested, processed, and plated onto BHI agar containing streptomycin. Burdens were enumerated 24-48 hours after plating. Data is cumulative of 2 independent experiments. (**D** and **E**) B6, Balb/c and Balb/cBy mice were infected with 2x10^9 CFU InlA^M *Lm* 10403s (**D**) or InlA^M *Lm* EGDe (**E**) and bacterial burdens were assessed at 5 dpi (**D**) while overall survival was assessed over 7 days (**E**). Data in (**D**) and (**E**) depicts 1 experiment each. The Mann-Whitney test was used to analyze the burdens in (**B**) and (**C**), while the bacterial burden in (**D**) was compared using one-way ANOVA with Kruskal-Wallis nonparametric test: * $p<0.05$; ** $p<0.01$; **** $p<0.0001$.

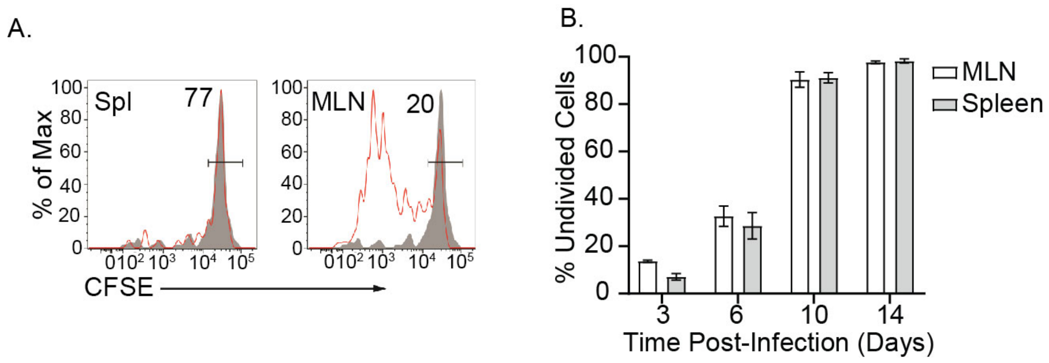

Figure S2. Antigen presentation in the MLN after foodborne *Lm* infection. (**A**) One day prior to infection, 1x10^6 CFSE-labelled congenic OT-I T cells were transferred into B6 mice. The following day, mice were infected with 2x10^9 CFU $InlA^M$ *Lm*-OVA 10403s. At 16 hpi, MLN and spleens were harvested and cultured for 72 hours. Proliferation was measured via CFSE dilution. (**B**) B6 mice were infected with 2x10^9 CFU $InlA^M$ *Lm*-OVA 10403s. At the noted timepoints, 1x10^6 CFSE-labelled congenic OT-I T cells were transferred into the mice. Cells from the MLN and spleen were harvested 3 days after each transfer, and proliferation was measured via CFSE dilution. Data are representative of at least 2 independent experiments with 3 mice per group.

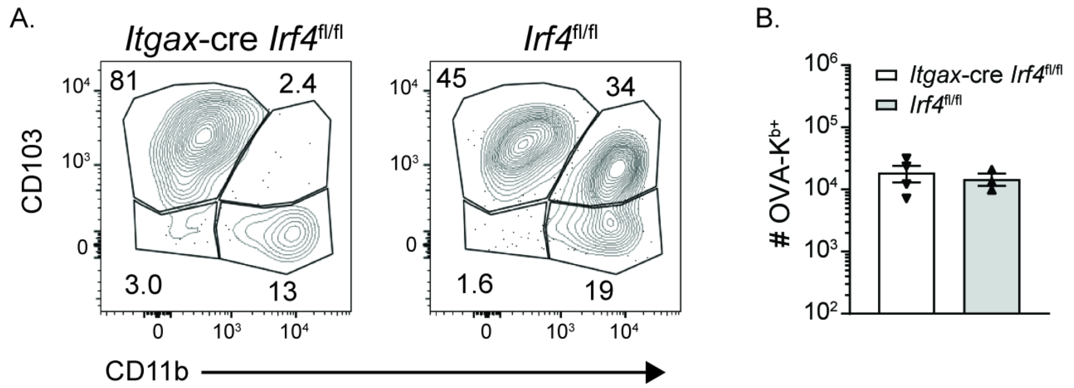

Figure S3. CD11b⁺ CD103⁺ DC are not critical for the induction of T cell responses in the MLN after foodborne InlA^{M} Lm infection. *Itgax*-cre *Irf4*$^{fl/fl}$ and littermate control mice were infected with $2x10^{9}$ CFU InlA^{M} *Lm*-OVA. At 9 dpi, MLN were harvested, processed, and stained for flow cytometry. (**A**) Representative flow plots of migratory DC subsets are gated on CD3^{-} CD19^{-} Ly6G^{-} Ly6C^{-} F4/80^{-} Ly6C^{-} MHCII^{hi} CD11c^{+} cells. (**B**) H-2K^{b} OVA tetramer staining was utilized to analyze antigen-specific CD8 T cell responses, and absolute numbers of OVA-specific CD8 T cells were quantified. Data are representative of 2 similar experiments.

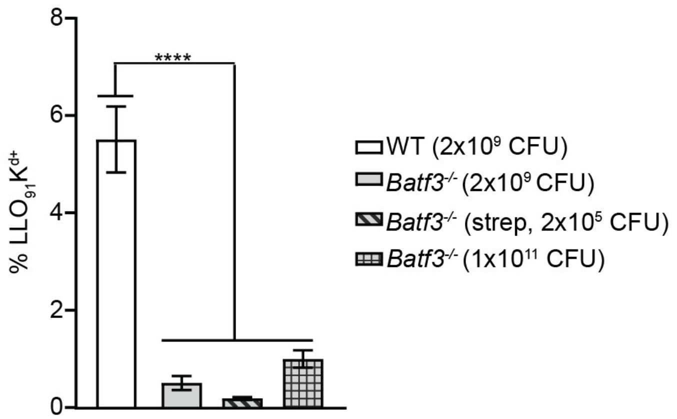

Figure S4. Restoration of pathogen burden does not rescue the IEL CD8 T cell response. Mice were or were not treated with 20 mg of streptomycin via oral gavage one day prior to infection as indicated. The following day, mice were foodborne infected with the indicated doses of *Lm*. At 9 dpi, IEL were harvested and processed for flow cytometry. LLO_{91}-K^d tetramer staining was utilized to assess *Lm*-specific CD8 T cell responses. Data are pooled from 5 experiments (n=5-22). One-way ANOVA with Bonferroni's correction was used to compare the groups. **** $p<0.0001$

Supplementary Table 1. Antibodies and reagents used.

Antigen	Clone	Conjugate	Company	Catalogue #	RRID #
α4β7	DATK32	PE	Biolegend	120606	AB_493267
α4β7	DATK 32	BV421	BD Biosciences	566294	AB_2739667
Fixable Viability Dye	N/A	eFlour780	eBioscience	65-0865-18	Not Available
Succinimidyl Ester	N/A	AF700	Life Technologies	A-20010	Not Available
CCR9	eBioCW-1.2	PE-Cy7	eBioscience	25-1991-82	AB_10854423
CD3ε	145-2C11	PE-Cy7	eBioscience	25-0031-82	AB_469572
CD3ε	145-2C11	PE-Dazzle	Biolegend	100348	AB_2564029
CD3ε	145-2C11	BV421	Biolegend	100336	AB_11203705
CD3ε	145-2C11	PerCP-Cy5.5	Biolegend	100328	AB_893318
CD3ε	145-2C11	PE	Biolegend	100308	AB_312673
CD4	GK1.5	BV711	Biolegend	100447	AB_2564586
CD4	GK1.5	PE-Cy7	Biolegend	100422	AB_11149488
CD8α	53-6.7	AF700	Biolegend	100730	AB_493703
CD8α	53-6.7	PE	Biolegend	100708	AB_312747
CD8α	53-6.7	BV650	Biolegend	100742	AB_2563056
CD11a	M17/4	BV510	BD Biosciences	747760	Not Available
CD11b	M1/70	PE-Cy7	Biolegend	101216	AB_312799
CD11b	M1/70	APC	Biolegend	101212	AB_312795
CD11b	M1/70	BV605	Biolegend	101257	AB_2565431
CD11b	M1/70	eFluor450	eBioscience	48-0112-82	AB_1582236
CD11c	N418	APC-ef780	eBioscience	47-0114-82	AB_1548652
CD11c	N418	PE-Cy7	Biolegend	117318	AB_493568
CD11c	N418	APC	Biolegend	117310	AB_313779
CD16/CD32	2.4G2	N/A	Bio X cell	BE0307	AB_2736987
CD19	6D5	PerCP-Cy5.5	Biolegend	115534	AB_2072925
CD19	6D5	PE-Cy7	Biolegend	115520	AB_313655
CD27	LG.3A10	PerCP-Cy5.5	Biolegend	124214	AB_2275577
CD44	IM7	APC-ef780	eBioscience	47-0441-82	AB_1272244
CD45	30-F11	AmCyan/V500	BD Biosciences	561487	AB_10697046
CD45	30-F11	BV510	Biolegend	103138	AB_2563061
B220 (CD45R)	RA3-6B2	FITC	Biolegend	103206	AB_31299
CD45.1	A20	Pacific Blue	Biolegend	110722	AB_492866
CD45.1	A20	PE	Biolegend	110708	AB_313497
CD45.2	104	APC-ef780	eBioscience	47-0454-82	AB_1272175
CD45.2	104	APC	Biolegend	109814	AB_389211
CD45.2	104	FITC	Biolegend	109806	AB_313443
CD64	X54-5/7.1	APC	Biolegend	139306	AB_11219391
CD80	16-10A1	FITC	Biolegend	104706	AB_313127
CD103	2E7	PE	Biolegend	121406	AB_1133989
CD127	A7R34	PE-Dazzle	Biolegend	135032	AB_2564217
CD127	A7R34	BV421	Biolegend	135024	AB_11218800
F4/80	BM8	PE-Dazzle	Biolegend	123146	AB_2564133
Goat α-Rabbit	α-Rabbit IgG H&L	AF488	Life Technologies	A-11070	AB_142134

Antigen	Clone	Conjugate	Company	Catalogue #	RRID #
IgG2a	C1.18.4	N/A	Bio X Cell	BE0085	AB_1107771
KLRG-1	2F1/KLRG1	PE-Cy7	Biolegend	138416	AB_2561736
KLRG-1	2F1/KLRG1	FITC	eBioscience	11-5893-82	AB_1311265
Ly6G	1A8	PerCP-Cy5.5	Biolegend	127616	AB_1877271
Ly6G	1A8	N/A	Bio X cell	BP0075-1	AB_1107721
MHC II	M5/114.15.2	Pacific Blue	Biolegend	107620	AB_493527
MHC II	M5/114.15.2	BV510	Biolegend	107636	AB_2734168
MHC II	M5/114.15.2	AF700	Biolegend	107622	AB_493727
Rabbit α-Lm	Difco Listeria O Antiserum Poly (Serotypes 1, 4)		BD Biosciences	DF2302-50-0	Not Available
SiglecF	E50-2440	BV421	BD Biosciences	562681	AB_2722581
